# Supplementary material for: The Inflammasomes Adaptor Protein PYCARD Is a Potential Pyroptosis Biomarker Related to Immune Response and Prognosis in Clear Cell Renal Cell Carcinoma
Source: Cancers (Basel). 2022 Oct 12;14(20):4992. doi: 10.3390/cancers14204992 (PMC9599636; doi:10.3390/cancers14204992)
Supplement: Supplementary file 1 [file cancers-14-04992-s001.zip › cancers-1900717-Supplementary Materials .pdf]

Supplementary Material

There are 6 supplementary figures and 4 supplementary tables.

Supplementary Tables Legends

Tables S1. PYCARD prognostic value in human cancers from PrognoScan database.

Tables S2. The details of tissue microarray and histochemistry scores.

Tables S3. The co-expression genes of PYCARD in KIRC from LinkedOmics database.

Tables S4. The GSEA results between PYCARD subgroups in KIRC cohort.

Supplementary Figures

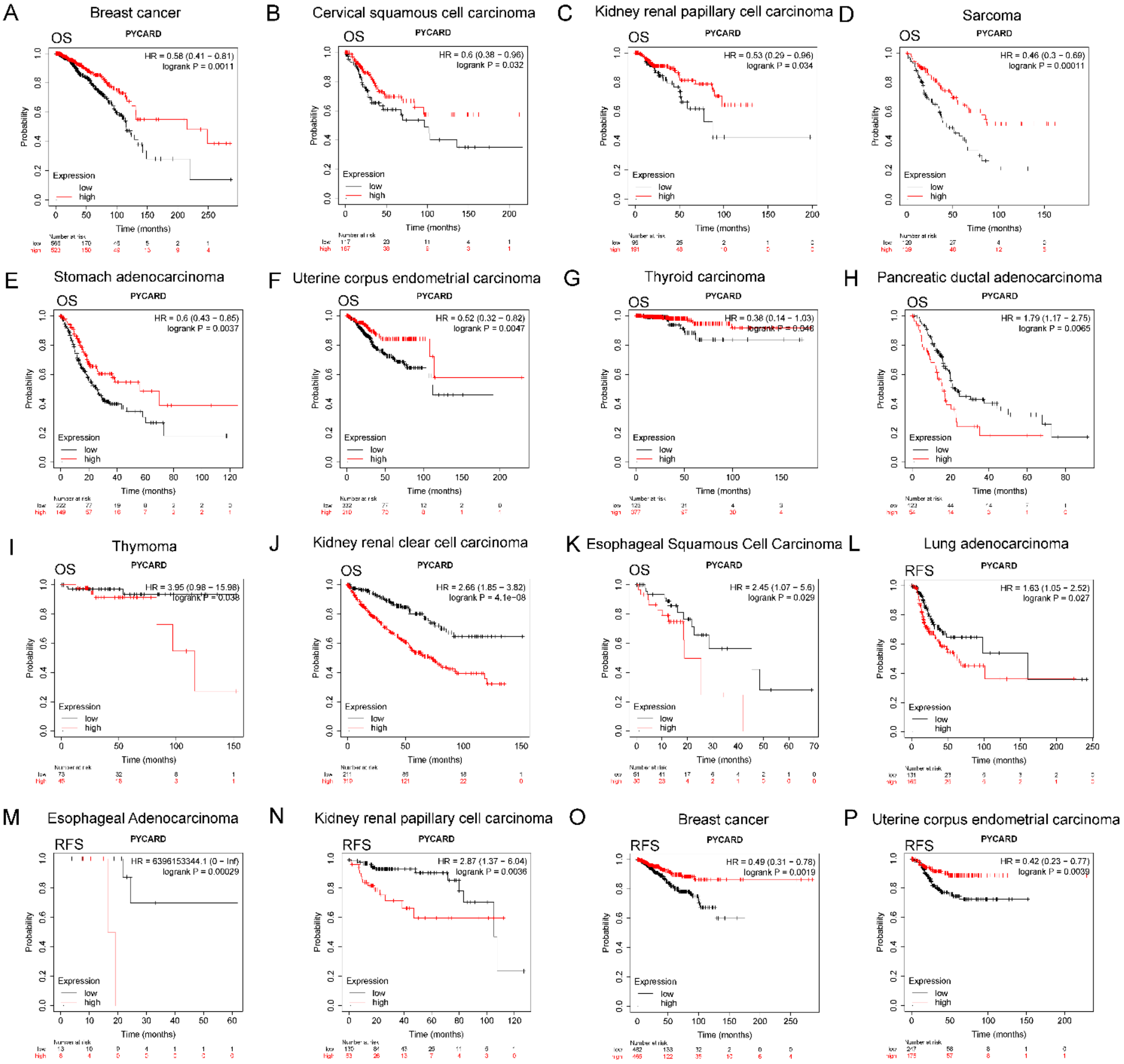

Figure S1. Kaplan-Meier survival analysis of high and low PYCARD expression from the Kaplan-Meier plotter database**.** Low PYCARD level was linked to worse OS in BRCA, CESC, KIRP, SARC, STAD, UCEC, and THCA respectively presented in A-G. High PYCARD level was linked to worse OS in PDAC, thymoma, KIRC, and esophageal squamous cell carcinoma presented in H-K. High PYCARD level was linked to worse RFS in LUAD, esophageal adenocarcinoma, and KIRP presented in L-N. High PYCARD level was linked to better RFS in BRCA, UCEC, and PCPG presented in O-Q. (OS, overall survival; RFS, relapse free survival)

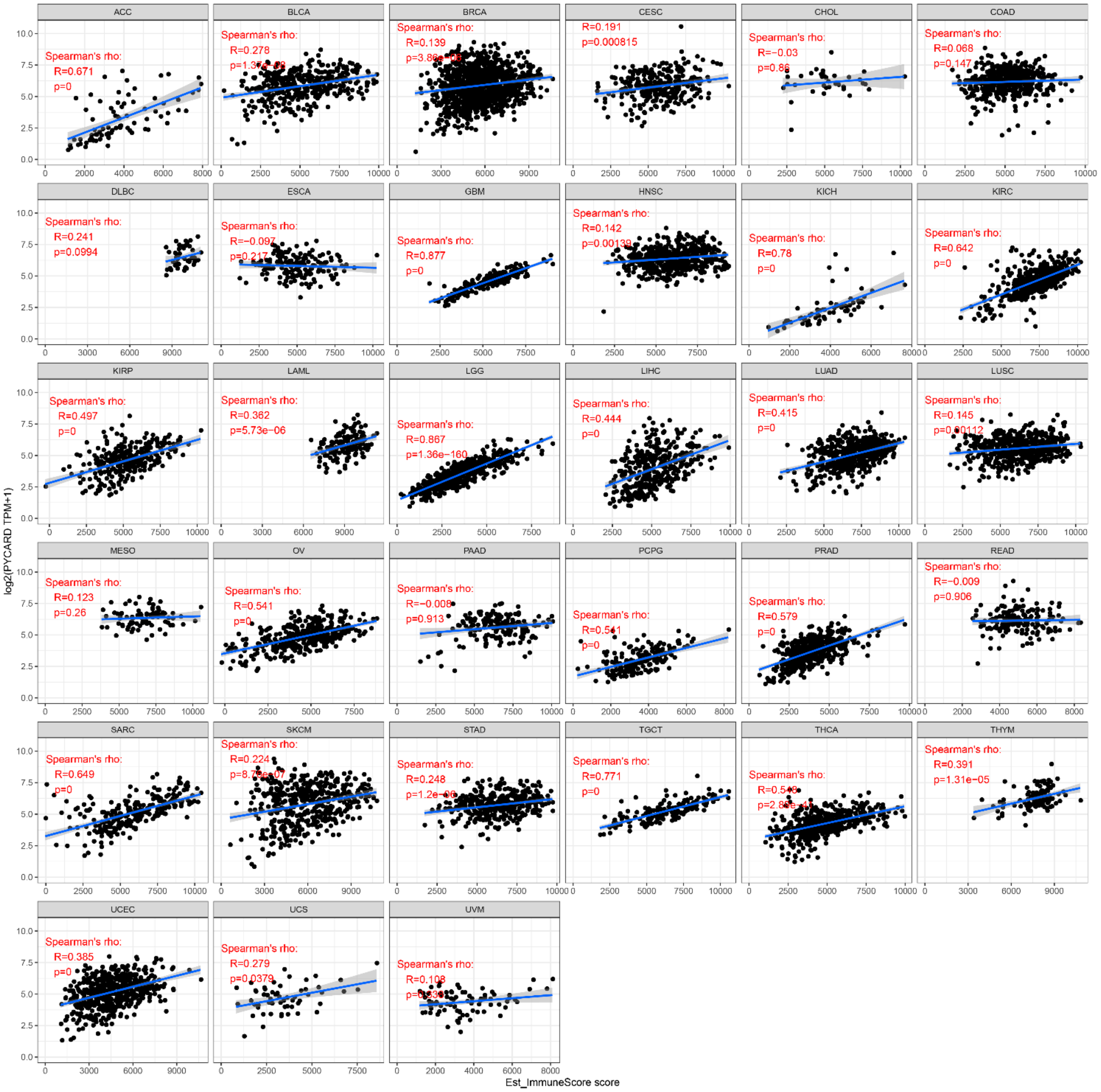

Figure S2. Correlations between PYCARD expression and ImmuneScore in pan-cancer analysis

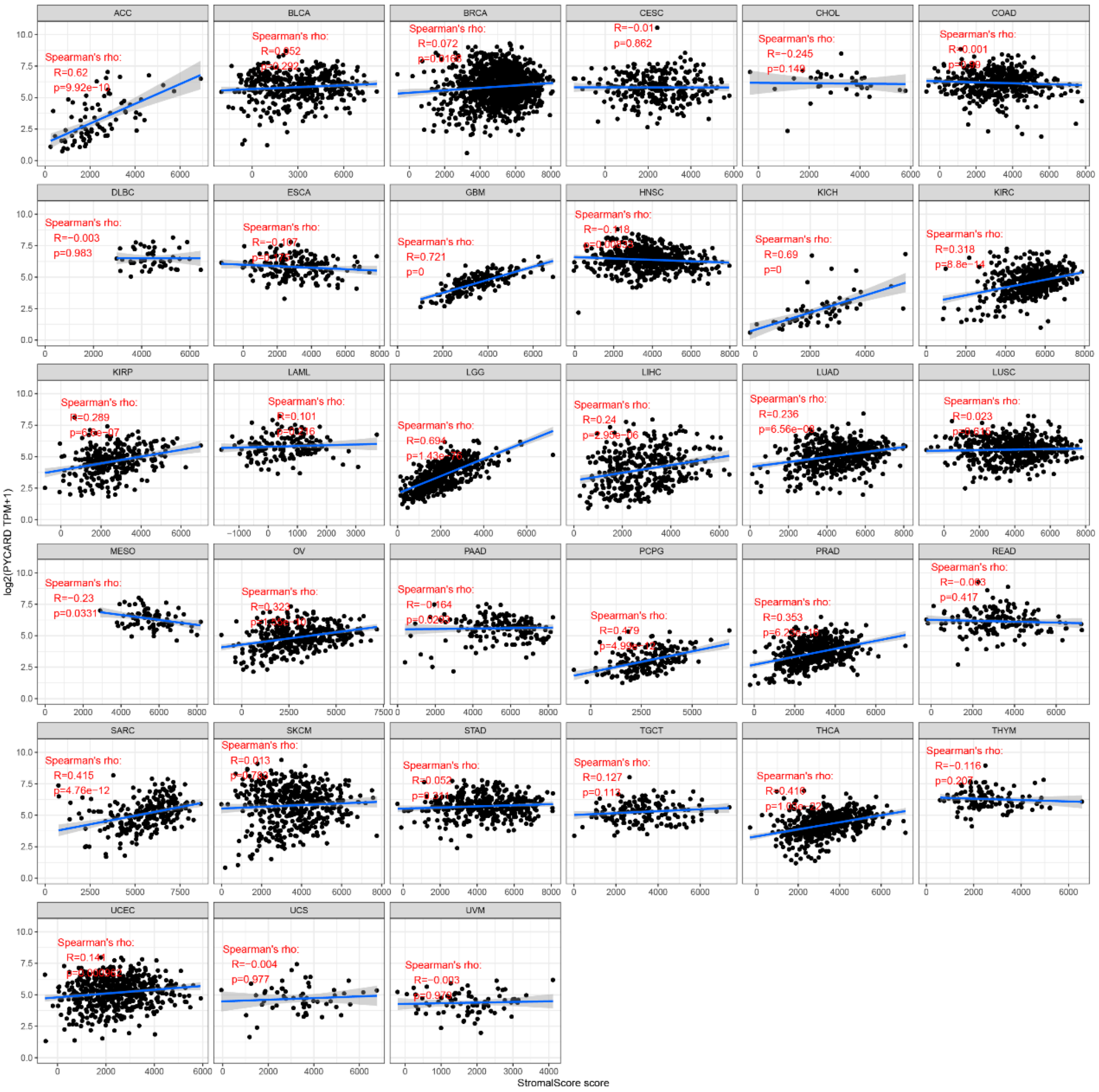

Figure S3. Correlations between PYCARD expression and StromalScore in pan-cancer analysis.

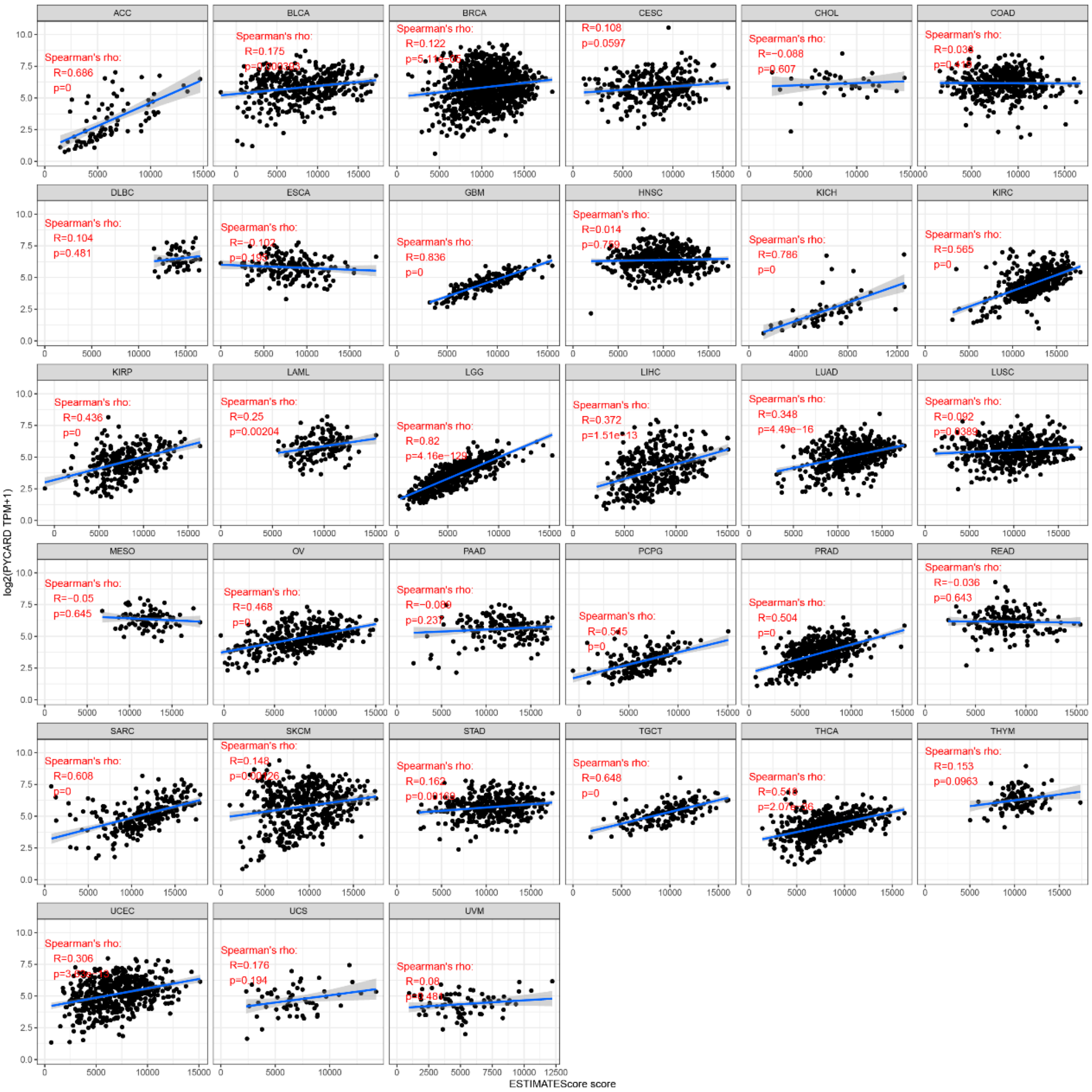

Figure S4. Correlations between PYCARD expression and ESTIMATEScore in pan-cancer analysis.

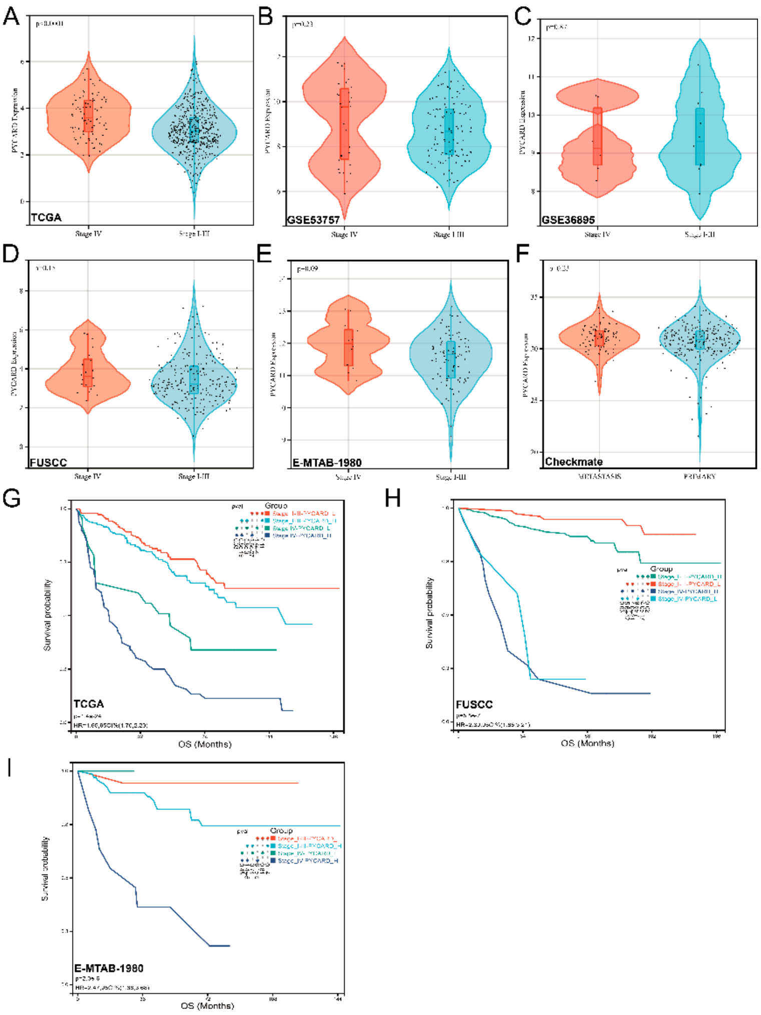

Figure S5. PYCARD expression and prognostic value in advanced ccRCC and early ccRCC. PYCARD expression differences between advanced ccRCC and early ccRCC were explored in TCGA (A), GSE53757 (B), GSE36895 (C), FUSCC Proteomic Cohort (D), E-MTAB-1980 (E) and Checkmate clinical trial (F) cohorts. Only in TCGA, PYCARD increased in advanced ccRCC patients than early ccRCC patients (P<0.0001). Survival subgroup analysis of PYCARD and stage classification in TCGA (G), FUSCC Proteomic Cohort (H), E-MTAB-1980 (I) cohorts.

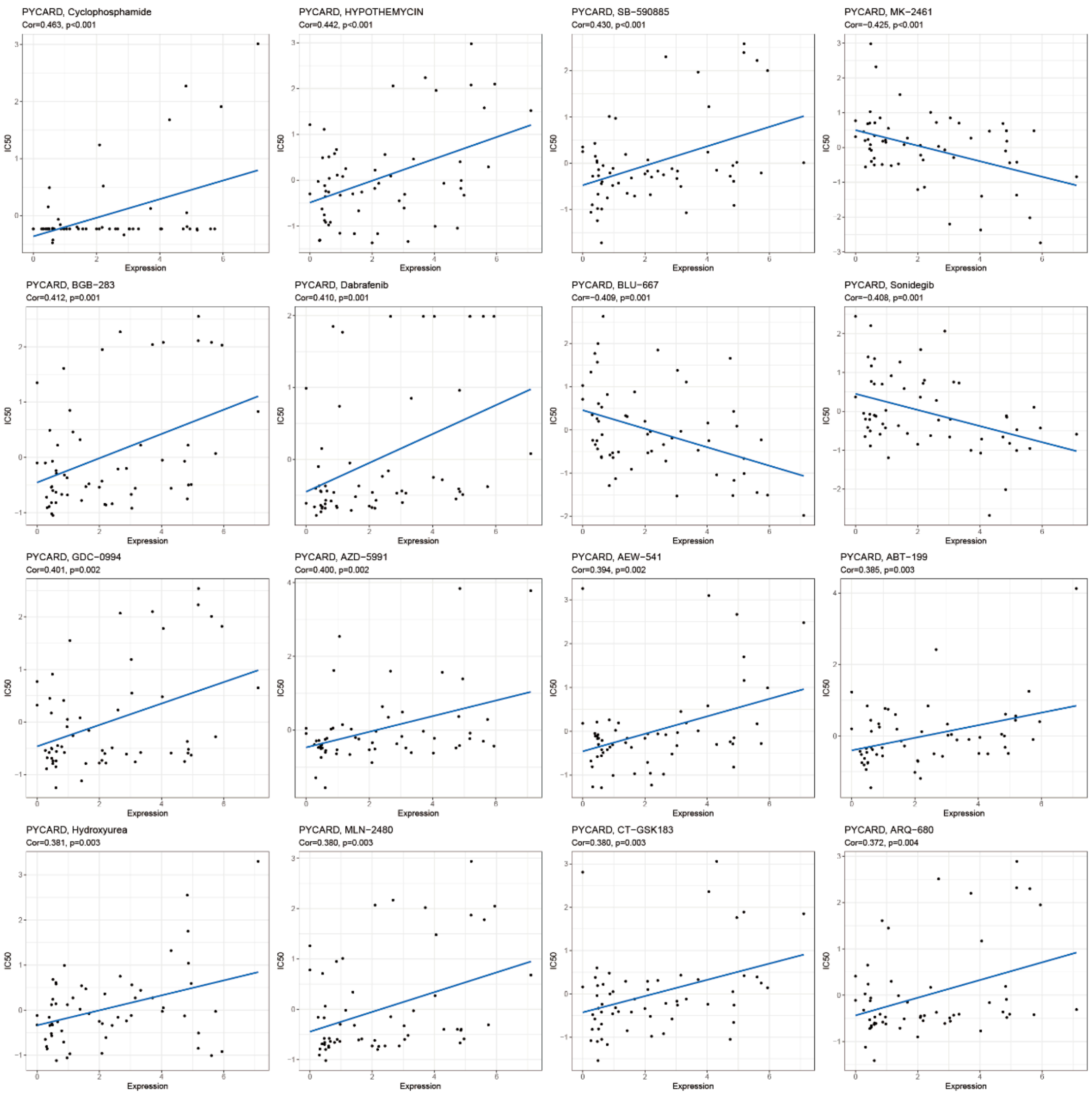

Figure S6. Associations between PYCARD expression and IC50 of the top sixteen-related drugs. PYCARD expression was positively correlated with cyclophosphamide, HYPOTHEMYCIN, SB-590885, BGB-283, dabrafenib, GDC-0994, AZD-5991, AEW-541, ABT-199, hydroxyurea, MLN-2480, CT-GSK183, and ARQ-680. In addition, PYCARD expression was negatively correlated with MK-2461, BLU-667, and sonidegib.
